# Venous thromboembolism prophylaxis in patients undergoing abdominal and pelvic cancer surgery: adherence and compliance to ACCP guidelines in DIONYS registry

**DOI:** 10.1186/s40064-016-3057-9

**Published:** 2016-09-13

**Authors:** Negib Geahchan, Melkart Basile, Maroon Tohmeh, on behalf of the DIONYS registry

**Affiliations:** 1 Faculty of Medicine, Saint Joseph University, Damascus street, Riad El Solh, P.O.Box 11-5076, Beirut, 1107 2180 Lebanon; 2 Faculty of Medical Sciences, Lebanese University, Beirut, Lebanon

**Keywords:** Neoplasms, Surgery, Venous thromboembolism, Anticoagulant, Guideline adherence, Patient compliance

## Abstract

**Background:**

Venous thromboembolism (VTE) is a major health care problem resulting in significant mortality, morbidity and increase in medical expenses. Patients with malignant diseases represent a high risk population for VTE. The American College of Chest Physicians (ACCP) proposed, since 1986, prophylaxis guidelines that are unequally respected in surgical practice.

**Methods:**

DIONYS is a multinational, longitudinal and non-interventional registry including patients having undergone abdominal or pelvic surgery for cancer in Latin America, Africa and the Middle East. Patients were evaluated with regard to VTE prophylaxis, during three consecutive visits, for their adherence to ACCP 2008 guidelines. Data were collected on type and duration of VTE prophylaxis, adherence to guidelines, and compliance with prescriptions, complications and possible reasons for omission of prophylaxis.

**Results:**

Between 2011 and June 2012, 921 adult patients were included and divided into abdominal (435), pelvic (390) and combined abdominal and pelvic surgery (96), 65.4 % being females. VTE prophylaxis was prescribed to 90 % of patients during hospitalization and to 28.3 % after hospital discharge. Prescriptions adhered to ACCP guidelines in 73.9 % of patients during hospitalization and 18.9 % after discharge. The reason of non-adherence was mainly the clinical judgment by the physician that the patient did not need a prophylaxis. The most commonly prescribed type of prophylaxis was pharmacological (low molecular weight heparin).

**Conclusion:**

A wide gap exists between VTE prophylaxis in daily practice and the ACCP 2008 guidelines, in abdominal and pelvic cancer surgery. A better awareness of surgeons is probably the best guarantee for improvement of VTE prophylaxis in surgical wards.

**Electronic supplementary material:**

The online version of this article (doi:10.1186/s40064-016-3057-9) contains supplementary material, which is available to authorized users.

## Background

Venous thromboembolism (VTE) is a disease entity that includes deep venous thrombosis (DVT) and pulmonary embolism (PE). It is recognized to be a major global health care problem resulting in significant mortality, morbidity and increase in medical expenses, all over the world. Its yearly incidence is between 117 and 160 per 100,000 in the general population, with a fatal PE rate of 50 per 100,000, making it the third most common circulatory disorder in the West (Nordström et al. 1992; Silverstein et al. 1998; Lindblad et al. 1991; Heit et al. 2000). The prevention of this frequent disease is mandatory, but it seems insufficiently done in the surgical practice of the majority of countries.

Surgery is a major risk factor of VTE. In the absence of effective VTE prophylaxis, the rate of asymptomatic DVT is reported to be 15–40 % in patients who undergo major abdominal or pelvic surgery resulting in 0.2–0.9 % rate of fatal PE event (Geerts et al. 2008; Mismetti et al. 2001). VTE is even more frequent in major orthopedic procedures, occurring in up to 60 % of patients (Krska 2012).

Factors affecting the risk of VTE in surgery include the following: extension and duration of surgeries, cancer, previous VTE, prolonged hospitalization, delayed immobilization, obesity, increasing age, type of anesthesia, postoperative infection, central venous catheter, prothrombic chemotherapeutic agents, genetic factors and trauma (Clarke-Pearson et al. 2003; Jacobson et al. 2005; Tateo et al. 2005; Martino et al. 2006; Srinivasan and Watzak 2012; Tagalakis et al. 2013).

Cancer is a well- documented risk factor for VTE, elevating its risk four to sevenfold when compared to the risk in cancer free patients (Heit et al. 2000). This association called Trousseau Syndrome is due to the key roles that angiogenesis and hemostasis play in the process of cancer genesis (Lecumberri et al. 2005). Although the risk of postoperative DVT is highest within the 1st week or two after surgery, VTE complications including fatal PE may occur later, with a peak reported between days 14 and 28 by some authors (Van Hemelrijck et al. 2013).

Extended VTE prophylaxis is a necessity to reduce the incidence of thrombotic complications in patients undergoing major surgeries. Scoring systems (Rogers, Caprini, IUAS) have been proposed (Caprini et al. 1991; Rogers et al. 2012) to classify patients in low, moderate, high and highest risk levels. The American College of Chest Physicians (ACCP) proposed guidelines based on these scoring systems to help in assessment of risk factors and to implement an appropriate use of VTE prophylaxis. These international guidelines were first published in 1986 and subsequently updated until the ninth edition in 2012 (Guyatt et al. 2012) by a panel of international experts. In case of abdominal or pelvic surgery for cancer, the guidelines recommend for high-VTE-risk patients who are not otherwise at high risk for major bleeding complications, a 4 week extended-duration pharmacologic prophylaxis with low molecular weight heparin (LMWH).

However, according to various sources, the VTE prophylaxis is far from optimal in current clinical practice. The adherence to international guidelines has been studied especially during the hospitalization period, and was shown to be low (Stratton et al. 2000; Kakkar et al. 2003; Wolff 2003; Amin et al. 2008).

Most of the developing countries do not have a homogenous management of venous thrombo-prophylaxis in agreement with international guidelines (Zeitoun et al. 2009; Ouro-Bang’na Maman et al. 2006; Arnaout et al. 2011; Bikdeli and Sharif-Kashani 2012; Mokhtari et al. 2011). For example, the ENDORSE study (Cohen et al. 2008) showed in 2006 that there were still low and heterogeneous rates of appropriate prophylaxis, notably across Latin America, Africa and Middle East countries (from 23 % in Venezuela to 78 % in Tunisia).

As the VTE risk continues after hospital discharge, further data are still needed on adherence and compliance of VTE prophylaxis with international guidelines concerning this post discharge period, regardless of the surgical procedure. Although such data have been obtained in major orthopedic interventions (Arcelus and Felicissimo 2013), they are still needed in abdominal and pelvic cancer surgery.

To answer these needs, a study has been performed within Latin America, Africa and Middle East countries: DIONYS (duration and adherence to International guidelines of venous thromboembolism prophylaxis in oncology patients undergoing a major abdominal or pelvic Surgery) is an international longitudinal, prospective and observational registry comparing real life administration of VTE prophylaxis to international ACCP 2008 guidelines, both during hospitalization and after discharge, in patients having undergone a major abdominal or pelvic surgery for malignant tumor.

## Methods

A longitudinal non-interventional registry have been designed for application in developing countries, namely Mexico and Venezuela (Latin America), Egypt (Africa), Saudi Arabia, Kuwait, United Arab Emirates (UAE), Syria and Lebanon (Middle East). The data from Kuwait and UAE were presented as a cluster because of limited sample size obtained in these 2 countries.

### Study design

The DIONYS registry was designed early in 2010 and started to be applied in 2011. It was conducted in accordance with the Declaration of Helsinki (1964), the International Conference on Harmonization Guidelines for Good Clinical Practice (ICH harmonized tripartite guideline), the good epidemiological practice (International epidemiology association guidelines 2009), and the local laws and regulations in all participating countries. The protocol was approved by local hospitals Ethics Committees and the informed consent forms were systematically signed by all included patients.

### Site and patient inclusion

A “master list” of private and public wards from each country was obtained and submitted for central randomization by the study monitor. Each ward was to recruit around 10 to 15 consecutive patients, the recruitment being done by the principal physician in the ward.

The inclusion criteria were: Male or female patient ≥18 years; abdominal or pelvic malignant tumor (colon, rectum, stomach, liver, pancreas, prostate, uterus, ovaries, bladder and kidney); major surgical procedure for this malignant tumor; informed consent signed prior to study entry.

The exclusion criteria were: An acute VTE event before the hospitalization within the last 6 months; long-term therapy with an anticoagulant agent for any reason (atrial fibrillation, previous VTE); life expectancy less than 3 months; current participation in a clinical trial; follow-up visit 6 weeks (at least 4 weeks) after surgery deemed to be impossible.

### Data collection

**Fig. 1:**
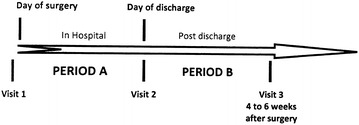
Study design of DIONYS

Data was collected using paper case report form (CRF) at Visit 1 (at admission to hospital), Visit 2 (at discharge), and Visit 3 (6 weeks, at least 4 weeks after surgery as per usual practice). Accordingly, period A (in hospital) and period B (post discharge) are distinguished in this study (Fig. 1). The computerized handling of the data by the study monitor after receipt of the CRFs could have generated additional requests to which the Investigator was obliged to respond by confirming or modifying the data questioned.

### Data management, review and validation

Source Data Verification (SDV) was performed on site, in 10 % minimum of the active sites, chosen at random in each country. This SDV was performed by a good clinical practice (GCP) qualified designated personnel in each country.

### Endpoints

The primary endpoint was the rate of in-hospital (period A) VTE prophylaxis, according to ACCP 2008 guidelines.

The secondary endpoints were the rate of VTE prophylaxis according to ACCP guidelines after hospital discharge (period B) and the reasons for lack of appropriate VTE prophylaxis during and after hospital discharge.

The information about these endpoints was collected in the questionnaires filled by the treating physician who was following his patient.

### Data statistical analyses

The Analysis Set consisted of the patients with a malignant tumor for whom major abdominal or pelvic surgery was documented, with complete information about age, sex and endpoints.

Generally, data was summarized and presented by type of surgery (abdominal, pelvic or abdominal + pelvic). Descriptive statistics were performed according to the type of criterion (quantitative or qualitative). Statistical tests were done (Chi square and Fisher exact test) with a type one error set at 0.05.

The influence of ward and patients’ characteristics on rates of period A, period B, and whole study VTE prophylaxis according to ACCP 2008 guidelines was studied by a univariate analysis for 14 criteria. For more details, consult the Additional file 1: appendix 1.

Criteria with a significance level of 0.15 or less were entered, consequently, in the multivariate analysis. A logistic regression model was implemented to explain the period A, the period B and the whole study adherences to ACCP 2008 guidelines. An ascending stepwise selection was used, with a significance level of 0.05. Egypt, the country with the highest number of patients included, was taken as reference.

## Results

DIONYS was planned to be performed in 5–10 Latin American, African, and Middle Eastern countries. The following countries or cluster of countries (Egypt; Saudi Arabia; Kuwait and UAE [clustered data]; Lebanon; Syria; Mexico; Venezuela) were initially selected, but Syria was excluded later on because of the unstable security situation.

**Fig. 2:**
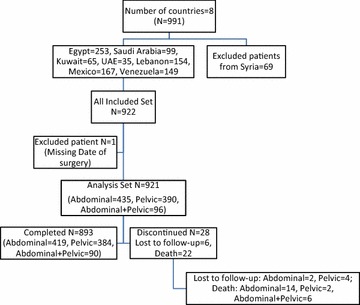
Overall participation status

The analysis set contained a total of 921 patients, included in 80 wards, and divided into 3 surgical groups: [Abdominal], [Pelvic], and [Abdominal + Pelvic]. The recruitment of patients started in 2011 and the cutoff date was June 29, 2012 (Fig. 2). Twenty-eight patients discontinued the study (3 %) and those lost to follow-up were 6 (0.7 %).

### Demographics and baseline characteristics

Table 1 shows the exact number of patients in the three groups with an equal distribution between abdominal and pelvic surgeries. The age average was approximately the same in the three surgical groups with predominance of the female gender in the [pelvic] and [abdominal + pelvic] groups, because of inclusion of gynecology wards.

**Table 1:** Patient demographics and characteristics by type of surgery

Patient characteristics	Statistics	Abdominal (N = 435)	Pelvic (N = 390)	Abdominal + pelvic (N = 96)	Total (N = 921)
Age (years)	N	435	390	96	921
	Mean (SD)	56.8 (14.1)	55.3 (14.3)	53.7 (12.9)	55.9 (14.1)
	Median	57.0	56.5	53.5	57.0
	Range	18–95	18–93	20–86	18–95
Gender	N	435	390	96	921
Male	N (%)	214 (49.2)	78 (20.0)	27 (28.1)	319 (34.6)
Female	N (%)	221 (50.8)	312 (80.0)	69 (71.9)	602 (65.4)
BMI (kg/m^2^)	N	415	379	93	887
	N missing	20	11	3	34
	Mean (SD)	27.03 (5.88)	28.81 (6.21)	28.35 (6.86)	27.93 (6.18)
	Median	26.20	28.00	27.20	27.30
	Range	10.4–67.0	15.3–66.7	14.3–59.6	10.4–67.0
≥One risk factor for VTE on top of surgery and cancer	N (%)	193 (44.4)	151 (38.7)	23 (24.0)	367 (39.8)
KPS score ≥80	N (%)	377 (86.6)	348 (89.3)	87 (90.7)	812 (88.2)
Laparotomy/open surgery	N (%)	387 (89.0)	362 (92.8)	91 (94.8)	840 (91.2)
Use of mechanical VTE prophylaxis	N (%)	182 (41.8)	195 (50.0)	36 (37.5)	413 (44.8)

*SD* standard deviation, *BMI* body mass index, *VTE* venous thromboembolism, *KPS* Karnofsky performance status

The mean BMI (Calle et al. 1999) was for the 3 groups of surgeries in the overweight category (mean in the total group: 27.93 kg/m^2^).

About 40 % of the included patients had at least one risk factor for VTE on top of surgery and cancer. Diabetes mellitus, moderate renal insufficiency, varicose veins and coronary artery diseases were on the top of the risk factors. A detailed description of these co-morbid conditions is given in Additional file 2: appendix 2.

The majority of the patients were in a good general status (KPS ≥ 80). All major abdominal organs, except liver, kidneys and prostate, were adequately represented in this study.

Surgery in a curative intent was done in the majority of cases (82.6 %), since few patients (11.9 %) had an advanced cancer disease (stage IV, M+) for whom surgical treatment was palliative. Chemotherapy and/or radiation therapy were used, either as neoadjuvant or during the study period, in 24.1 % of cases. These treatments represent also a specific VTE risk factor associated to cancer. The detailed data of the patients’ cancer characteristics are found in Additional file 3: appendix 3.

### Rates of utilization of VTE prophylaxis: Period A, period B and throughout study period

To the majority of patients (90.3 %), at least one type of VTE prophylaxis was prescribed during the whole study period. A combination of mechanical and pharmacological prophylaxis was prescribed in 46.6 % while 40.7 % were given pharmacological treatment alone and 3 % only mechanical prophylaxis (Table 2).

**Table 2:** Number of patients for whom a VTE prophylaxis was prescribed by period of time

n (%)	Abdominal (N = 435)	Pelvic (N = 390)	Abdominal + Pelvic (N = 96)	Total (N = 921)
Period of intake				
Before surgery	1 (0.2)	4 (1.0)	0	5 (0.5)
From surgery to hospital discharge	387 (89.0)	357 (91.5)	85 (88.5)	829 (90.0)
None	48 (11.0)	33 (8.5)	11 (11.5)	92 (10.0)
Mechanical only	10 (2.3)	18 (4.6)	0	28 (3.0)
Pharmacological only	173 (39.8)	149 (38.2)	50 (52.1)	372 (40.4)
Mechanical + Pharmacological	204 (46.9)	190 (48.7)	35 (36.5)	429 (46.6)
After hospital discharge	104 (23.9)	126 (32.3)	31 (32.3)	261 (28.3)
None	331 (76.1)	329 (67.7)		660 (71,7)
Mechanical only	8 (1.8)	44 (9.1)		52 (5.6)
Pharmacological only	90 (20.7)	105 (21.6)		195 (21.2)
Mechanical + Pharmacological	6 (1.4)	8 (1.6)		14 (1.5)
During the whole study (from surgery to post-hospital discharge)	387 (89.0)	360 (92.3)	85 (88.5)	832 (90.3)
None	48 (11.0)	30 (7.7)	11 (11.5)	89 (9.7)
Mechanical only	10 (2.3)	18 (4.6)	0	28 (3.0)
Pharmacological only	173 (39.8)	152 (39.0)	50 (52.1)	375 (40.7)
Mechanical + Pharmacological	204 (46.9)	190 (48.7)	35 (36.5)	429 (46.6)

*VTE* venous thromboembolism

The mechanical methods of prophylaxis included the graduated elastic compression (GEC) (38.4 %), the intermittent pneumatic compression (IPC) (15 %) and bandages (9.3 %). The pharmacological methods of prophylaxis used mainly LMWH (86.6 %), while UFH was prescribed only in 3 % and OAC in 0.5 %. Among LMWH, enoxaparin was the most frequently proposed (93.8 %).

Before surgery, VTE prophylaxis was exceptionally prescribed to the included patients (0.5 %). A great difference in rates of VTE prophylaxis was noted between periods A and B (90 versus 28.3 %). Whereas the two treatment modalities are frequently associated in the period A, in the latter, the mechanical methods are often neglected in favor of the pharmacological drugs.

### Omission of VTE prophylaxis in period A

Table 3 details the reasons for omission of prescription of VTE prophylaxis during hospitalization. The main reason invoked by the physician was the low risk for VTE of the patient. Economic and logistic reasons were scarcely reported.

**Table 3:** Reason for no VTE prophylaxis during hospitalization

	Abdominal (N = 435)	Pelvic (N = 390)	Abdominal + Pelvic (N = 96)	Total (N = 921)
No VTE prophylaxis prescribed during hospitalization	48 (11.0)	30 (7.7)	11 (11.5)	89 (9.7)
Reason				
Low risk of VTE	32 (66.7)	15 (50.0)	8 (72.7)	55 (61.8)
No drug available at hospital	2 (4.2)	8 (26.7)	0	10 (11.2)
Bleeding	6 (12.5)	3 (10.0)	0	9 (10.1)
Economic reason	4 (8.3)	2 (6.7)	0	6 (6.7)
Omission from principal investigator	0	2 (6.7)	3 (27.3)	5 (5.6)
Nausea/vomiting	2 (4.2)	0	0	2 (2.2)
Pulmonary embolism	1 (2.1)	0	0	1 (1.1)
Other	1 (2.1)	0	0	1 (1.1)

*VTE* venous thromboembolism

### Omission of VTE prophylaxis in period B

The Additional file 4: appendix 4 indicates that the major reason for non-extension of VTE prophylaxis after hospital discharge was the clinical judgment of the physician that the patient did not need an extension of this prophylaxis (91.4 %). All other reasons were of no significant importance.

### Adherence of prescribed prophylaxis to guidelines

**Fig. 3:**
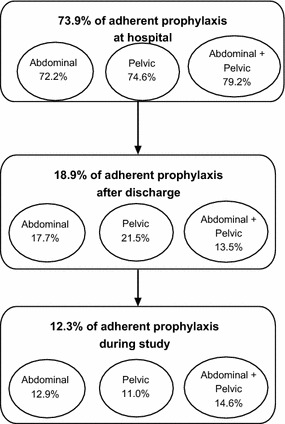
Percentage of VTE prophylaxis adherent to ACCP 2008 guidelines at each period

In this study, the overall adherence to ACCP 2008 guidelines (concerning the type of prophylaxis, the drug dosage and the duration of treatment) was very low (12.3 %) and this was mainly due to a non-respect of these guidelines in period B. A high adherence rate (73.9 %) was noted during period A (Fig. 3).

Following univariate results, country, type of hospital, total number of interventions per year and VTE prophylaxis protocol/policy were criteria found to be significantly influencing the period A adherence rate. The period B adherence rate was also influenced, in addition to these criteria, by the type of surgery, the type of anesthesia, the disease status and the use of mechanical VTE prophylaxis. In the whole study rates, the same criteria were retained in addition to the presence of at least one medical history of VTE and hemorrhagic risk factor, the disease status and the current treatment.

The country, the type of hospital and the number of interventions per year were kept in the final multivariate logistic regression for the whole study and period A. In period B, the multivariate analysis retained the type of anesthesia and the use of mechanical VTE prophylaxis, in addition to the country. Lebanon was the most adherent country to VTE prophylaxis in accordance with ACCP guidelines 2008 (74.1 %), followed by Mexico (8.6 %), Egypt (8 %), the Cluster countries (4 %), Saudi Arabia (3.4 %) and Venezuela (1.7 %).

### Reasons for patients’ non- compliance to prescription

Of 918 patients who were supposed to receive VTE prophylaxis as per the ACCP 2008 guidelines, 253 patients (27.6 %) only took the correct treatment. The great majority did not receive this treatment because it was not prescribed by the physician. The non-compliance to a correct prophylaxis according to ACCP guidelines was a rare event (1.1 %). Table 4 shows the reasons for not complying with the prescribed treatment.

**Table 4:** Reasons for non compliance of patients to the prescribed treatment

	Statistics	Abdominal	Pelvic	Abdominal + pelvic	All
Analysis set	N	435	390	96	921
Missing data	Nmiss	3	0	0	3
Prophylactic treatment should be prescribed	N	432	390	96	918
*Prohylactic treatment prescribed and correctly taken*	*n (%)*	*101 (23.4)*	*122 (31.3)*	*30 (31.3)*	*253 (27.6)*
Not taken	n (%)	4 (0.9)	5 (1.3)	1 (1.0)	10 (1.1)
If no, reason					
Economic reason	n (%)	2 (0.5)	1 (0.3)	0	3 (0.3)
Definitive prophylaxis discontinuation for medical reason	n (%)	0	2 (0.5)	0	2 (0.2)
No pharmacy/No drug available at pharmacy	n (%)	0	1 (0.3)	0	1 (0.1)
Discomfort	n (%)	1 (0.2)	0	0	1 (0.1)
Omission	n (%)	0	1 (0.3)	0	1 (0.1)
Other	n (%)	0	0	1 (1.0)	1 (0.1)
Discomfort + Definitive prophylaxis discontinuation for medical reason	n (%)	1 (0.2)	0	0	1 (0.1)
No or partial re-imbursement by Health system	n (%)	0	0	0	0
Self-injection not possible	n (%)	0	0	0	0
No-one to perform the injection	n (%)	0	0	0	0
Missing	Nmiss	0	0	0	0
*Prophylactic treatment not prescribed and not taken*	*n (%)*	*327 (75.7)*	*262 (67.2)*	*65 (67.7)*	*654 (71.2)*
Unknown	n (%)	0	1 (0.3)	0	1 (0.1)

### Complications during the study

When considering the whole study period, 202 patients (22.3 %) presented at least one complication. The most frequent events were wound infection, re-intervention/revision and septicemia. It should be noted that bleeding causing hematoma on surgical site and VTE, two complications directly related to VTE prophylaxis, were particularly rare events.

The possible association between the occurrence of complications and the adherence to ACCP 2008 guidelines during the whole study period was studied by a univariate analysis and showed no significant relation (Additional file 5: appendix 5).

Death occurred in 22 patients out of 921 (2.4 %). Pulmonary embolism was diagnosed as cause of death in 5 cases (0.54 %).

### Treatment duration

Among LMWH, enoxaparin was the most frequently prescribed for VTE prophylaxis (93.8 %). In 188 patients out of 921, enoxaparin was the only agent used for anti VTE prophylaxis (Additional file 6: appendix 6). The duration of treatment had a mean of 24.3 days.

## Discussion

Because of the high risk of VTE in major surgical interventions, the ACCP proposed in 1986 a series of guidelines, recommending or suggesting an active strategy aiming to prevent these undesirable events (ACCP-NHLBI 1986). These guidelines were systematically and periodically reviewed by ACCP, until the 8th edition in 2008 (Hirsh et al. 2008) and the 9th edition in 2012 (Guyatt et al. 2012). DIONYS, realized in the time period of 2010–2011, adopted the recommendations of 2008 and aimed to evaluate the hospitals and the physician’s adherence to these guidelines as well as the compliance of patients with their prescriptions. The results of DIONYS registry are still applicable today because the 9th edition of the ACCP guidelines does not differ dramatically from the 8^th^ edition, mainly regarding the abdominal and pelvic surgery for cancer (Guyatt et al. 2012).

Although the majority of patients received any type of VTE prophylaxis during the in-hospital period, only 79 % of them had prescriptions adherent to the ACCP 2008 guidelines. The situation was totally different in the post-discharge period when even less than 30 % received any prophylaxis, and only 19 % were adherent to guidelines. These results pinpoint the great lack of appropriate VTE prophylaxis in cancer surgical patients in the developing countries, mainly after discharge from hospital.

DIONYS has been the first study to evaluate the adherence to the ACCP guidelines exclusively in cancer surgical patients. All other published data concerned medical and/or surgical patients, including a variable number of cancer cases.

Many publications have reported different rates of adherence to the ACCP guidelines. In North America, a study by Yu (Yu et al. 2007) on 123,304 hospitalized patients reported an overall 13.3 % in-hospital adherence rate with ACCP 1996 guidelines. Omission of prophylaxis was the primary reason for this low rate, followed by an inadequate duration of prophylaxis. Amin, in 2008, reported in the USA (Amin et al. 2008) that about 73 % of hospitalized cancer patients did not receive adequate VTE prophylaxis according to ACCP 2004 guidelines. This inappropriateness of treatment was equally distributed on medical and surgical patients, and was essentially due to unawareness of the physicians. These results were in agreement with other North American studies reporting similar weak adherence rates, both in medical (Goldhaber and Tapson 2004; Kahn et al. 2007) and surgical (Kakkar et al. 2003) wards.

Around the world, Bikdeli (Bikdeli and Sharif-Kashani 2012), in a recent review, evaluated the VTE prophylaxis status of various patient subsets, and found important discrepancies between the different regions. Similarly, the ENDORSE study (Cohen et al. 2008), which was a cross sectional survey assessing adherence to ACCP guidelines in 32 countries over 5 continents, revealed a rate of adherence to the guidelines in the risky surgical patients varying between 23 and 58 % in the countries included in the DIONYS study and between 0.2 and 92 % worldwide, with the highest rates recorded in Western Europe. The “prophylaxis appropriateness” assessed in the ENDORSE study included only the type of VTE prophylaxis prescribed for in-patients, irrelevant of the duration of prescription. Accordingly, it is likely that the overall adherence rate was overestimated in this study.

The subject was addressed by similar studies in the developing countries. The AVAIL ME study was a Middle Eastern comprehensive evaluation of VTE prophylaxis, conducted to assess the status of anticoagulation practices in seven countries (Taher et al. 2011). Of 2266 patients, 82.9 % were eligible for prophylaxis according to the ACCP 2004 guidelines. Fifty-one percent obtained some form of VTE prophylaxis, but only 37.8 % according to the ACCP guidelines. The study included medical and surgical patients and revealed that adherence to ACCP guidelines was 44 % in the surgical group, higher than that of the medical group. Later on, the AVAIL ME Extension study (Mokhtari et al. 2011) included ten Middle Eastern and Asian countries and defined the rate of patients receiving appropriate prophylaxis according to ACCP 2008 guidelines. Sixty-eight percent of patients were surgical, and cancer surgery constituted 14.1 % of these patients. Application of VTE prophylaxis guidelines was found only in 32 % of all patients. Thirty-nine percent of all surgical patients followed correctly the guidelines. The rate of patients who did not require or had contraindications to VTE prophylaxis received it in 78 and 66 % respectively. Similar to the ENDORSE study; AVAIL ME was cross-sectional using a 1-day assessment, so VTE prophylaxis duration appropriateness couldn’t be assessed.

In Lebanon, a multi-centric prospective chart review study of 840 patients (Zeitoun et al. 2009), showed appropriate VTE prophylaxis in about 65 % of patients at low risk, 30 % of patients at moderate risk and 61 % of patients at high risk with a total of 58.5 % of adherence to guidelines. Thirty-five (46.7 %) of the cancer patients received suitable prevention, whereas 40 (53.4 %) were improperly managed.

In a multi-centric Brazilian study regrouping four hospitals (Deheinzelin et al. 2006), it was demonstrated that 29 % of the highest surgical VTE risk patients were not prescribed prophylaxis correctly while 42 % of low-risk patients were over treated. In Africa, a survey among surgeons about their practice habits was conducted in Nigeria (Akinmoladun et al. 2007) and Togo (Ouro-Bang’na Maman et al. 2006) and showed that respectively 47.5 and 6 % of them used prophylaxis routinely in major surgeries, according to their own evaluation of the risk.

However, all these publications did not tackle the adherence to guidelines in the post-discharge period, limiting their figures mainly to the in-hospital period. Only recently, the DEIMOS registry (Arcelus and Felicissimo 2013), a study similar in its methodology to the DIONYS registry and covering ten countries in Latin America, Africa and the Middle East, compared the real life VTE prophylaxis received by major orthopedic patients with the 2008 ACCP guidelines during the complete post-surgery period: 85 % of patients were prescribed a VTE prophylaxis during hospitalization according to guidelines and 63.4 % after hospital discharge.

In DIONYS registry, the main reason for not prescribing VTE prophylaxis according to ACCP guidelines in the in-hospital period was a medical decision that the patients were at low risk of VTE (69 %), followed by the absence of treatment at the hospital, the occurrence of bleeding, some economic reasons and the omission of prescription by the surgeon. In the post-discharge period, the reason was simply a medical judgment that prophylaxis was not useful anymore (91 %). Several factors were studied by a logistic regression model looking for their eventual influence on the omission of appropriate VTE prophylaxis. The country, the type of hospital and the number of interventions per year and per hospital were retained for the in-hospital period. The country, the type of anesthesia and the use of mechanical VTE prophylaxis were the risk factors in the post-discharge period.

Although there was a widespread belief that VTE is low in cancer patients and that VTE treatment is less effective in cancer surgery (Amin et al. 2008), Kakkar et al. (Kakkar and Williamson 1999) reported that cancer patients undergoing surgery have twice the risk of postoperative VTE and more than three times the fatal risk of PE than patients who undergo surgery for benign condition.

Post-operative VTE, independently from other factors, has been found to be a poor prognostic factor in cancer patients even after complete surgical resection (Auer et al. 2012). In the ENOXACAN II and other studies (Bergqvist et al. 2002; Schmeler et al. 2013; Huo and Muntz 2009; Osborne et al. 2008), LMWH prophylaxis for 4 weeks after surgery for abdominal or pelvic cancer was found safe and it significantly reduces the incidence of venographically demonstrated thrombosis and the likelihood of symptomatic VTE (OR: 0.22), as compared with LMWH prophylaxis for 1 week. In the Aristos study (Agnelli et al. 2006), five risk factors, including advanced cancer, were identified as risk of VTE after surgery. Lecumberri (Lecumberri et al. 2005) reported some oncologic trials showing that adjunction of LMWH to antineoplastic treatments had a beneficial effect on survival independently of all other cancer prognostic factors.

The barriers to appropriate VTE prophylaxis were comprehensively analyzed by Bikdeli (Bikdeli and Sharif-Kashani 2012) who found many reasons for this inadequate care. Resulting from these barriers, VTE remains a major health problem in surgery: in three surgical services in the USA, over the 10-year period since initial publication of the guidelines, VTE occurred in 0.46 % of surgical patients and the incidence increased gradually over the years despite 84 % of partial or complete adherence to guidelines (Shackford et al. 2008).

Compliance with the VTE prophylaxis prescriptions, particularly in the post-discharge period, should be distinguished from the adherence to ACCP guidelines, since the latter depends on a physician decision while the former is patient-dependent. The non -compliance of patients with prescriptions, in the DIONYS registry, was only 1.1 % emphasizing once again that the problem of inappropriateness of VTE prophylaxis resides in the physician behavior. Bikdeli (Bikdeli and Sharif-Kashani 2012) analyzed also this condition and identified, as reasons for it, the injection site pain, bruises, systemic complications, skin breaks and local discomfort.

Prophylactic doses of anticoagulation have not been associated with hemorrhagic complications. In our study, only 0.7 % of the patients had a major bleeding. Our data are in line with the earlier studies that showed major bleeding occurring in 1.2 % in cancer surgery as well as in outpatient setting (Kahn et al. 2012; Rasmussen et al. 2006). In DIONYS, VTE related death occurred in 0.54 % of the patients. Other studies showed that death secondary to VTE was between 0 and 0.8 % (Agnelli et al. 2006; Kahn et al. 2012; Rasmussen et al. 2006).

The method of VTE prophylaxis used in DIONYS was mainly pharmacological and the most prescribed agent during the whole study was enoxaparin (81.3 %). It is universally admitted that the VTE prophylaxis may be pharmaceutical, mechanical or combined. If there is contraindication to anticoagulation or recurrence of PE despite optimal anticoagulation, vena cava filters (VCF) may be considered (Farge et al. 2013).

Among pharmaceutical agents, LMWH are the preferred agents for the initial and long term VTE prophylaxis in patients with neoplastic disease, based on randomized clinical trials. They are given once daily, do not usually require routine monitoring but should be given with precaution in renal insufficiency patients (Streiff 2009; Streiff 2011). In cancer patients, LMWHs compared to oral warfarin are at least as effective. Less osteoblast activation is observed in response to LMWH exposure (Deitcher 2003).

DIONYS has both strengths and limitations. It was a large observational, prospective and multinational registry, conducted on patients from seven developing countries across three continents. It left the physicians free to prescribe whatever VTE prophylaxis they found appropriate, thus studying real life application of prophylaxis according to ACCP guidelines. Another strong point was that, even though some studies concerning the adherence to ACCP guidelines were conducted previously, DIONYS, to our knowledge, was the first multinational study, conceived in these countries and taking into consideration not only the type, but also the duration of VTE prophylaxis, and its extension to the post-discharge period, in abdominal and pelvic surgery for cancer.

However, in DIONYS, there was a possibility of a selection bias in case the master-list of hospitals was not complete in all countries. Another relative limitation was the fact that this registry studied the adherence of VTE prophylaxis to ACCP 2008 guidelines, and since that time, newer 2012 guidelines had emerged, showing the need for newer studies. DIONYS only assessed the application of VTE prophylaxis guidelines in abdominal and pelvic cancer surgery, thus the results may not be applicable to patients undergoing other cancer surgeries.

In conclusion, DIONYS revealed that a large gap remains, at least in the developing countries, between real life practices of VTE prophylaxis in cancer surgery and the international ACCP guidelines. The non-adherence to guidelines could be improved by arranging continuous education programs for the medical team, increasing their awareness to the problem. Prophylaxis protocols should be established and implemented at both the hospital and the national levels trying to promote the recommended policies in the real life practice.

### DIONYS investigators

Registry Scientific Committee: Négib Elias Geahchan. The list of investigators who recruited patients in the DIONYS registry is available as Additional file 1: Appendix 1.

## Additional files

10.1186/s40064-016-3057-9 Adherence to accp 2008 guidelines critiria of possible influence on adherence. Univariate and multiple analysis.
10.1186/s40064-016-3057-9 Co-Morbid conditions and risk factors.
10.1186/s40064-016-3057-9 Oncologic baseline characteristics.
10.1186/s40064-016-3057-9 Reasons for the absence of prescription of VTE prophylaxis after hospital discharge.
10.1186/s40064-016-3057-9 Complications during the whole study period.
10.1186/s40064-016-3057-9 Duration of treatment with Enoxaparin in the 3 groups of surgery.

## Abbreviations

VTE: venous thromboembolism
DVT: deep venous thrombosis
PE: pulmonary embolism
IUAS: International Union of Angiology Consensus Statement
ACCP: American College of Chest Physicians
LMWH: low molecular weight heparin
ENDORSE: epidemiologic international day for the evaluation of patients at risk for venous thromboembolism in the acute hospital care setting
CRF: case report form
SDV: source data verification
GCP: good clinical practice
CI: confidence interval
BMI: body mass index
KPS: Karnofsky performance status
M+: Metastatic
GEC: Graduated elastic compression
IPC: intermittent pneumatic compression
UFH: unfractionated heparin
OAC: oral anticoagulants
AVAIL ME: assessment for VTE management in hospital-middle east
DEIMOS: duration and adherence to international guidelines of venous thromboembolic prophylaxis after major orthopedic surgery
HIT: heparin induced thrombocytopenia
VCF: vena cava filters
N: numbers
SD: standard deviation
UAE: United Arab Emirates
