# Supplementary material for: Venous thromboembolism prophylaxis in patients undergoing abdominal and pelvic cancer surgery: adherence and compliance to ACCP guidelines in DIONYS registry
Source: Springerplus. 2016 Sep 13;5(1):1541. doi: 10.1186/s40064-016-3057-9 (PMC5020030; doi:10.1186/s40064-016-3057-9)
Supplement: Supplementary file 1 — 10.1186/s40064-016-3057-9 Adherence to accp 2008 guidelines critiria of possible influence on adherence. Univariate and multiple analysis. [file 40064_2016_3057_MOESM1_ESM.docx]

**Online appendix 1**

**ADHERENCE TO ACCP 2008 GUIDELINES**

**CRITIRIA OF POSSIBLE INFLUENCE ON ADHERENCE**

UNIVARIATE AND MULTIPLE ANALYSIS

1. country / cluster of countries,
2. type of hospital/clinic (Private, Public or both),
3. teaching hospital (Yes or No),
4. number of interventions performed per year (<100, from 100 to 400 or >400),
5. thrombo-prophylaxis protocol/policy (Yes or No),
6. disease status (Local disease (stade I-II), Locally advanced (stade III), Advanced disease (stade IV / M+)),
7. current cancer treatment (At least one current treatment, No treatment),
8. Karnofsky Performance Status (KPS) (<80, ≥80),
9. type of surgery (Abdominal, Pelvic, Abdominal + Pelvic),
10. use of mechanical VTE prophylaxis during the surgery (Yes, No),
11. type of anesthesia (General, Regional),
12. post-operative analgesia (Yes, No),
13. medical history related to VTE and hemorrhagic risk factors (Yes, No),
14. age (<50, [50;60[, ≥60), sex (Male, Female ) and BMI (<25, [25;30[, ≥30).

| VTE, Venous thromboembolism; BMI, Body mass index; M+, Metastatic |
| --- |
