# Supplementary material for: Venous thromboembolism prophylaxis in patients undergoing abdominal and pelvic cancer surgery: adherence and compliance to ACCP guidelines in DIONYS registry
Source: Springerplus. 2016 Sep 13;5(1):1541. doi: 10.1186/s40064-016-3057-9 (PMC5020030; doi:10.1186/s40064-016-3057-9)
Supplement: Supplementary file 2 — 10.1186/s40064-016-3057-9 Co-Morbid conditions and risk factors. [file 40064_2016_3057_MOESM2_ESM.docx]

**Online appendix 2**

**Co-Morbid conditions and risk factors**

|  | **Statistics** | **Abdominal (N=435)** | **Pelvic (N=390)** | **Abdominal + Pelvic (N=96)** | **Total (N=921)** |
| --- | --- | --- | --- | --- | --- |
| At least one medical history / venous thromboembolism and hemorrhagic risk factor by case | N | 193 (44.4) | 151 (38.7) | 23 (24.0) | 367 (39.8) |
| . Diabetes mellitus | n (%) | 71 (16.3) | 74 (19.0) | 13 (13.5) | 158 (17.2) |
| . Other | n (%) | 75 (17.2) | 56 (14.4) | 11 (11.5) | 142 (15.4) |
| . Moderate renal insufficiency | n (%) | 40 (9.2) | 34 (8.7) | 3 (3.1) | 77 (8.4) |
| . Varicose veins | n (%) | 25 (5.7) | 16 (4.1) | 2 (2.1) | 43 (4.7) |
| . Coronary Artery Disease | n (%) | 18 (4.1) | 16 (4.1) | 1 (1.0) | 35 (3.8) |
| . Hepatic disease | n (%) | 10 (2.3) | 5 (1.3) | 0 | 15 (1.6) |
| . Chronic Heart Failure | n (%) | 6 (1.4) | 2 (0.5) | 1 (1.0) | 9 (1.0) |
| . Respiratory insufficiency | n (%) | 2 (0.5) | 3 (0.8) | 1 (1.0) | 6 (0.7) |
| . Stroke | n (%) | 3 (0.7) | 2 (0.5) | 0 | 5 (0.5) |
| . Hormonal therapy | n (%) | 2 (0.5) | 3 (0.8) | 0 | 5 (0.5) |
| . Peripheral arterial disease | n (%) | 2 (0.5) | 2 (0.5) | 0 | 4 (0.4) |
| . Venous insufficiency | n (%) | 2 (0.5) | 1 (0.3) | 0 | 3 (0.3) |
| . Bleeding disorders | n (%) | 3 (0.7) | 0 | 0 | 3 (0.3) |
| . History of venous thrombo-embolism | n (%) | 0 | 1 (0.3) | 0 | 1 (0.1) |
