# Supplementary material for: Venous thromboembolism prophylaxis in patients undergoing abdominal and pelvic cancer surgery: adherence and compliance to ACCP guidelines in DIONYS registry
Source: Springerplus. 2016 Sep 13;5(1):1541. doi: 10.1186/s40064-016-3057-9 (PMC5020030; doi:10.1186/s40064-016-3057-9)
Supplement: Supplementary file 3 — 10.1186/s40064-016-3057-9 Oncologic baseline characteristics. [file 40064_2016_3057_MOESM3_ESM.docx]

**Online appendix 3**

**Oncologic baseline characteristics**

| Cancer characteristics | **Statistics** | **[Abdominal] (N=435)** | **[Pelvic] (N=390)** | **[Abdominal + Pelvic] (N=96)** | **Total (N=921)** |
| --- | --- | --- | --- | --- | --- |
| Site of primary malignant tumor | Effective N (%) | 435 | 390 | 96 | 921 |
| - Colon |  | 193 (44.4) | - | 17 (17.7) | 210 (22.8) |
| - Stomach |  | 111 (25.5) | - | - | 111 (12.0) |
| - Pancreas |  | 44 (10.1) | - | - | 44 (4.8) |
| - Uterus |  | - | 155 (39.7) | 13 (13.5) | 168 (18.2) |
| - Ovaries |  | - | 101 (25.9) | 25 (26.0) | 126 (13.7) |
| - Rectum |  | - | 89 (22.8) | 20 (20.8) | 109 (11.8) |
| - Bladder |  | - | 14 (3.6) | 15 (15.6) | 29 (3.1) |
| - Others |  | 87 (20.0) | 31 (8.0) | 6 (6.4) | 124 (13.4) |
| Disease status | Effective N | 435 | 390 | 96 | 921 |
| Local disease (stade I - II) | N (%) | 185 (42.5) | 200 (51.3) | 36 (37.5) | 421 (45.7) |
| Locally advanced (stade III) | N (%) | 189 (43.4) | 151 (38.7) | 50 (52.1) | 390 (42.3) |
| Advanced disease (stade IV / M+) | N (%) | 61 (14.0) | 39 (10.0) | 10 (10.4) | 110 (11.9) |
| Type of surgery | Effective N | 435 | 390 | 96 | 921 |
| - Curative | N (%) | 333 (76.6) | 345 (88.5) | 83 (86.5) | 761 (82.6) |
| Current cancer treatment | Effective N | 435 | 390 | 96 | 921 |
| - Radiotherapy | N (%) | 6 (1.4) | 16 (4.1) | 6 (6.3) | 28 (3.0) |
| - Chemotherapy | N (%) | 67 (15.4) | 50 (12.8) | 18 (18.8) | 135 (14.7) |
| - Radiotherapy + Chemotherapy | N (%) | 15 (3.4) | 38 (9.7) | 6 (6.3) | 59 (6.4) |
| - Other | N (%) | 78 (17.9) | 47 (12.0) | 18 (18.8) | 143 (15.5) |
| - No treatment | N (%) | 269 (61.8) | 239 (61.3) | 48 (50.0) | 556 (60.4) |
