# Supplementary material for: Venous thromboembolism prophylaxis in patients undergoing abdominal and pelvic cancer surgery: adherence and compliance to ACCP guidelines in DIONYS registry
Source: Springerplus. 2016 Sep 13;5(1):1541. doi: 10.1186/s40064-016-3057-9 (PMC5020030; doi:10.1186/s40064-016-3057-9)
Supplement: Supplementary file 4 — 10.1186/s40064-016-3057-9 Reasons for the absence of prescription of VTE prophylaxis after hospital discharge. [file 40064_2016_3057_MOESM4_ESM.docx]

**Online appendix 4**

**Reasons for the absence of prescription of VTE prophylaxis
after hospital discharge**

|  | **[Abdominal] (N=435)** | **[Pelvic] (N=390)** | **[Abdominal + Pelvic] (N=96)** | **Total (N=921)** | **p-value*** |
| --- | --- | --- | --- | --- | --- |
| **No VTE prophylaxis prescribed after discharge: N, (Percentage)** | 326 (74.9) | 262 (67.2) | 65 67.7) | 653 (70.9) |  |
| **Reason**: N and %** |  |  |  |  |  |
| Extended prophylaxis medically not justified | 294 (90.2) | 246 (93.9) | 57 (87.7) | 597 (91.4) | 0.147 |
| Other | 32 (9.8) | 16 (6.1) | 8 (12.3) | 56 (8.6) |  |
| Economic reason | 21 (6.4) | 11 (4.2) | 2 (3.1) | 34 (5.2) |  |
| Dead | 8 (2.5) | 1 (0.4) | 6 (9.2) | 15 (2.3) |  |
| Bleeding | 1 (0.3) | 1 (0.4) | 0 | 2 (0.3) |  |
| Omission from principal investigator | 0 | 1 (0.4) | 0 | 1 (0.2) |  |
| No-one to perform injection at home | 1 (0.3) | 0 | 0 | 1 (0.2) |  |
| Refusal from patient to take VTE prophylaxis | 0 | 1 (0.4) | 0 | 1 (0.2) |  |
| No drug available at the site | 1 (0.3) | 0 | 0 | 1 (0.2) |  |
| Pulmonary embolism | 0 | 1 (0.4) | 0 | 1 (0.2) |  |
| *:  p-value from Chi² or Fisher exact test **: The physician filling the report was allowed to mention more than 1 cause for lack of VTE prophylaxis VTE = Venous Thromboembolism N = number % = percentage reported to upper numerical figures | | | | | |
