# Supplementary material for: Venous thromboembolism prophylaxis in patients undergoing abdominal and pelvic cancer surgery: adherence and compliance to ACCP guidelines in DIONYS registry
Source: Springerplus. 2016 Sep 13;5(1):1541. doi: 10.1186/s40064-016-3057-9 (PMC5020030; doi:10.1186/s40064-016-3057-9)
Supplement: Supplementary file 5 — 10.1186/s40064-016-3057-9 Complications during the whole study period. [file 40064_2016_3057_MOESM5_ESM.docx]

**Online appendix 5**

**Complications during the whole study period**

|  | **Statistics** | **[Abdominal]** | **[Pelvic]** | **[Abdominal + Pelvic]** | **All** |
| --- | --- | --- | --- | --- | --- |
| **Analysis Set** | **N** | **435** | **390** | **96** | **921** |
| **Data available for Complications** | **N** | **426** | **384** | **96** | **906** |
| . No complication | N (%) | 323 (75.8) | 312 (81.3) | 69 (71.9) | 704 (77.7) |
| . Occurrence of complication | N (%) | 103 (24.2) | 72 (18.8) | 27 (28.1) | 202 (22.3) |
| **Complications** |  |  |  |  |  |
| . Bedsore | N (%) | 1 (0.2) | 0 | 1 (1.0) | 2 (0.2) |
| . Wound infection | N (%) | 62 (14.6) | 47 (12.2) | 15 (15.6) | 124 (13.7) |
| . Other infection | N (%) | 6 (1.4) | 11 (2.9) | 3 (3.1) | 20 (2.2) |
| . Re-intervention/revision | N (%) | 14 (3.3) | 9 (2.3) | 3 (3.1) | 26 (2.9) |
| . Myocardial infarction | N (%) | 0 | 0 | 0 | 0 |
| . Stroke | N (%) | 0 | 0 | 0 | 0 |
| . Cognitive function impairment | N (%) | 0 | 0 | 0 | 0 |
| . Other | N (%) | 34 (8.0) | 15 (3.9) | 5 (5.2) | 54 (6.0) |
| . Deep Venous Thrombosis | N (%) | 2 (0.5) | 3 (0.8) | 0 | 5 (0.6) |
| . Post-Thrombotic Syndrome | N (%) | 0 | 0 | 0 | 0 |
| . Pulmonary Embolism | N (%) | 3 (0.7) | 2 (0.5) | 1 (1.1) | 6 (0.7) |
| . Hematoma on surgical site | N (%) | 5 (1.2) | 1 (0.3) | 0 | 6 (0.7) |
| . Septicemia | N (%) | 13 (3.1) | 4 (1.0) | 3 (3.1) | 20 (2.2) |
| . Occlusive complication | N (%) | 5 (1.2) | 1 (0.3) | 2 (2.1) | 8 (0.9) |
