# Supplementary material for: Venous thromboembolism prophylaxis in patients undergoing abdominal and pelvic cancer surgery: adherence and compliance to ACCP guidelines in DIONYS registry
Source: Springerplus. 2016 Sep 13;5(1):1541. doi: 10.1186/s40064-016-3057-9 (PMC5020030; doi:10.1186/s40064-016-3057-9)
Supplement: Supplementary file 6 — 10.1186/s40064-016-3057-9 Duration of treatment with Enoxaparin in the 3 groups of surgery. [file 40064_2016_3057_MOESM6_ESM.docx]

**Online appendix 6**

**Duration of treatment with Enoxaparin in the 3 groups of surgery**

|  | **Statistics** | **[Abdominal[** | **[Pelvic]** | **[Abdominal + Pelvic]** | **All** |
| --- | --- | --- | --- | --- | --- |
| Analysis Set | N | 88 | 86 | 14 | 188 |
| Treatment duration (days) | N | 78 | 81 | 12 | 171 |
|  | Nmiss | 10 | 5 | 2 | 17 |
|  | Mean (SD) | 26.5 (12.4) | 21.7 (8.9) | 27.8 (8.2) | 24.3 (10.8) |
| Treatment duration after discharge (days) | N | 78 | 81 | 12 | 171 |
|  | Nmiss | 10 | 5 | 2 | 17 |
|  | Mean (SD) | 16.4 (9.4) | 14.2 (8.1) | 17.8 (7.0) | 15.5 (8.7) |
